# Glioblastoma‐activated pericytes support tumor growth via immunosuppression

**DOI:** 10.1002/cam4.1375

**Published:** 2018-02-25

**Authors:** Isadora F. G. Sena, Ana E. Paiva, Pedro H. D. M. Prazeres, Patrick O. Azevedo, Luiza Lousado, Sujit K. Bhutia, Alla B. Salmina, Akiva Mintz, Alexander Birbrair

**Affiliations:** ^1^ Department of Pathology Federal University of Minas Gerais (UFMG) Belo Horizonte MG Brazil; ^2^ Department of Life Science National Institute of Technology Rourkela Odisha India; ^3^ Department of Biochemistry Krasnoyarsk State Medical University Krasnoyarsk Russia; ^4^ Department of Radiology Columbia University Medical Center New York New York

**Keywords:** Glioblastoma, immunosuppression, microenvironment, pericytes

## Abstract

Glioblastoma multiforme is the most common and aggressive primary brain tumor, with an extremely poor prognosis. The lack of detailed knowledge about the cellular and molecular mechanisms involved in glioblastoma development restricts the design of efficient therapies. A recent study using state‐of‐art technologies explores the role of pericytes in the glioblastoma microenvironment. Glioblastoma‐activated pericytes develop an immunosuppressive phenotype, reducing T‐cell activation through the induction of an anti‐inflammatory response. Strikingly, pericytes support glioblastoma growth in vitro and in vivo. Here, we describe succinctly the results and implications of the findings reported in pericytes' and glioblastomas' biology. The emerging knowledge from this study will be essential for the treatment of brain tumors.

## Introduction

Glioblastoma multiform is the most common brain tumor in humans with very aggressive progression 1. Despite the fact that glioblastoma is a rare tumor (<10 per 100,000 people in the world), its poor prognosis with survival rate of 14–15 months after diagnosis makes it a global medical problem 2. Tumor progression in the case of glioblastoma is very fast and poorly controlled with traditional chemotherapy. The dismal prognosis is a direct result of the fact that standard therapies fail to eradicate residual or infiltrating cells that reside adjacent to and infiltrate normal brain tissue 3. The complexity of glioblastoma development and sensitivity to common therapeutic protocols is caused by several factors, including heterogeneity of glial cells within the tumor and appearance of different subclones, high level of vascularization due to excessive neoagiogenesis, impaired integrity of the blood–brain barrier, variability in the intracerebral location, multiple routes for cell migration, existence of tumor‐controlled microenvironment with glioblastoma‐associated stem cells, or perivascular cells affecting tumor growth in a complex and poorly predicted manner 4, 5. Although several therapies are currently in use, including small‐molecule kinase inhibitors, antivascular endothelial growth factor monoclonal antibodies, immune checkpoint inhibitors, epigenetic modulators, and transfer of lethal genes into tumor cells, glioblastoma treatment is still the most challenging task in clinical oncology 2, 6. Establishment of the tumor microenvironment is a key mechanism of acquiring self‐controlled and autonomous growth in the tumors. The lack of a detailed knowledge about the cellular and molecular mechanisms mediating glioblastoma progression restricts the design of effective antitumoral treatments. Cerebral microvessels have higher pericytes/endothelial cells ratio (10–30‐fold) than other tissues 7, therefore, contribution of blood vessels, specifically pericytes, to the establishment of glioblastoma microenvironment has attracted interest in the recent years.

Pericytes were defined, more than a century ago, as a population of contractile cells with long projections encircling the blood vessel walls 8, 9, 10. The limited capacity of microscopy, before the 21st century, resulted in the notion of the pericyte acting merely as a vascular supporting cell 11. Recently, several modern technologies, such as confocal microscopy and transgenic mice models, led to rapidly expanding insights into the varying functions, sometimes unexpected, of pericytes in physiology and pathology. Pericytes interact with astrocytes to regulate the maintenance of the blood–brain barrier 12, 13, 14. They also participate in vascular development, maturation, and remodeling, as well as contributing to its normal architecture and permeability 15, 16, 17, 18, 19. Pericytes regulate the blood flow 20, and recent studies showed that pericytes can function as stem cells, generating several other cell types, including neural cells 3, 21, 22, 23, 24, 25, 26, 27, 28, 29, 30, 31.

## Pericytes Affect Glioblastoma Immune Microenvironment

Interestingly, immune regulation also depends of pericytes. The reader is referred to excellent reviews that discuss these pericytes roles in detail 32, 33. In brief, pericytes play immune functions by regulating lymphocytes activation 34, 35, 36, 37, by attracting innate leukocytes that exit through the sprouting vessels 38, by contributing to the clearance of toxic cellular byproducts, as pericytes possess phagocytic activity 39, and by affecting blood coagulation 40, 41, 42, 43, 44, 45, 46, 47. Nonetheless, little is known about the pericytes' roles in the brain tumor microenvironment. Now, in a recent article in *Oncotarget*, Valdor and colleagues show that the conditioning by brain tumor cells induces immunosuppression by pericytes 48. The authors discovered that, after activation by glioblastoma tumor cells, pericytes secrete high levels of anti‐inflammatory cytokines, such as IL‐10 and TGF*β*, while they do not produce proinflammatory cytokines, such as IL‐1, IL‐23, and IL12, which could be produced in other conditions by brain pericytes 49. This immunomodulatory phenotype in brain pericytes requires glioblastoma tumor cell–pericyte interaction 48. The glioblastoma‐activated pericytes downregulate the expression of costimulatory surface membrane molecules, such as CD80, CD86, and the major histocompatibility complex class II. Additionally, Valdor and colleagues revealed that in response to the interaction with glioblastoma cancer cells, T‐cell activation by pericytes is impaired 48. Normal brain pericytes activated proliferation and IL‐2 production by T cells. In contrast, in the presence of glioblastoma‐activated pericytes, T cells showed defective proliferation and IL‐2 production.

**Figure 1:**
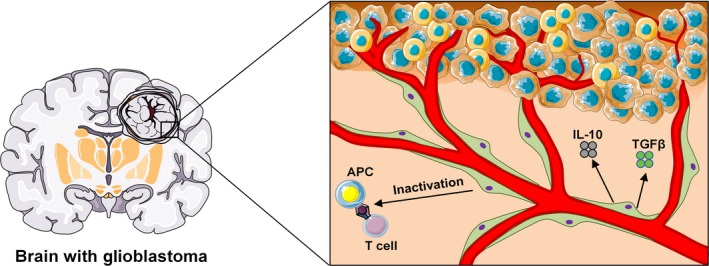
Influence of pericytes in the glioblastoma microenvironment. Pericytes are associated to cerebral blood vessels. The study of Valdor and colleagues now reveals a novel very important function of pericytes during glioblastoma development 48. Glioblastoma‐activated pericytes increase the expression of anti‐inflammatory molecules, such as IL‐10, TGFβ, and MHC‐II, favoring immunosuppression and tumor growth. With the appearance of state‐of‐art modern techniques technologies 38, future studies will reveal in detail all cellular components and their interaction with tumor cells in the glioblastoma microenvironment.

Strikingly, pericytes promoted glioblastoma growth in vitro and in vivo. This work provides a novel possible central cellular population to be pharmacologically targeted in patients with brain tumors. Here, we discuss the findings from this study and evaluate recent advances in our understanding of the roles of pericytes in the glioblastoma microenvironment (Fig. 1).

## Perspectives/future Directions

The conclusions from this study claiming the immunosuppressive role of pericytes in the glioblastoma microenvironment are based on observations made in animals that received transplants of pericytes previously propagated in culture, or on experiments performed utilizing pericytes grown in vitro. Note that artificial conditions and high concentration of mitogens that characterize cell culture systems may induce some characteristics in pericytes that may not be shared by the corresponding endogenous brain pericytes in vivo under pathophysiological conditions 24, 50. Transgenic mouse models constitute currently the most reliable strategy to study the behavior of any given cell population in vivo 51. These mice have been widely applied to study cell types within diverse tissues microenvironments. The ability to delete single genes in specific cell types in adult mice has allowed us to answer specific questions regarding the roles of different cell populations in the regulation of several physiologic and pathologic processes 52, 53, 54, 55, 56, 57. In the brain tumor microenvironment, the exact identities of all cells that play important roles in the pathogenesis of this condition remain uncertain 58, 59. Valdor et al. (2017) now proposed that pericytes develop an immunosuppressive phenotype in response to interaction with glioblastoma cells 48. Nevertheless, important anti‐inflammatory cytokines have not been conditionally deleted from brain pericytes, so there is no direct evidence that pericytes are the only/main functionally important source of those cytokines to produce the immunosuppression during glioblastoma progression. This issue may be addressed, owing to technological breakthroughs, by analyzing the effect of genetic ablation of specific cytokines such as IL‐10 and TGF*β* in brain pericytes on the glioblastoma development. Moreover, the generation of IL10‐ or TGF*β*‐floxed mice to be crossed with pericyte‐specific inducible CreER driver, such as NG2‐CreERT2 59, will allow us to specifically delete these cytokines in pericytes. In addition to studies in genetically modified mouse models, transcriptomic and single cell analysis represents fundamental tools that will help us understand the roles of pericytes within the brain tumor microenvironment.

Valdor and colleagues used a xenografted mouse model of glioblastoma, in which immunocompetent mice received human glioblastoma cancer cells 48. Human glioblastoma cells cause some level of immune reaction in immunocompetent mice, simply because these cells derive from another specie. Having the immune rejection of the host as a limiting factor, the use of other mouse models for glioblastoma that do not require transplantation may allow to study endogenous pericytes at different stages of glioblastoma development. A spontaneous mouse model of glioblastoma has been engineered, Nf1/Trp53 mutant mice develop endogenous glioblastoma 60 and could be used in future studies of pericytes biology during brain tumor progression.

## Pericytes Heterogeneity

Valdor and colleagues examine pericytes as a homogeneous cell population in their work. Nevertheless, pericytes have been shown to be heterogeneous regarding their phenotype, distribution, origin, marker expression, and function 62. Pericytes associated with different blood vessel types differ in their morphology, markers, and function 60, 63, 64, 65, 66, 67, 68, 69. At least two pericyte subpopulations have been described in the brain. Type‐1 and type‐2 pericytes were distinguished based on the presence or absence of Nestin‐GFP expression 70. Another group identified two brain pericyte subsets based on their heterogeneous expression of desmin, *α* smooth muscle actin, and glutamate aspartate transporter (Glast) 71. Also, ATP sensitive potassium channel Kir6.1 only labels a subset of pericytes in the brain 72. Interestingly, only type‐2 pericytes participate in tumoral angiogenesis 29. Thus, whether only a fraction of pericytes promote immunosuppression during glioblastoma growth still needs to be explored. It would be interesting to evaluate whether distinct pericytes' subsets behave differently after exposure to glioblastoma tumor cells. Furthermore, the precise identity of glioblastoma cancer cells is poorly defined. It seems that these tumors comprise of heterogeneous malignant cells with subclones and glioblastoma stem cells. Whether these malignant cell populations interact differently with pericytes should be explored in future studies.

A central nervous system pericyte subpopulation have been recently shown to take part in the formation of the scar tissue after brain lesion, which is a major obstacle to neuronal regeneration in patients with this condition 70, 73. The lesion induces an increase in the number of a pericyte subtype, while the number of the other pericyte subtype did not change 70. Interestingly, some brain pericytes dissociate from endothelial cells, losing contact with the blood vessels after the lesion. It will be interesting to understand whether the pericytes that participate in scar formation are the same or differ from the ones that promote immunosuppression in the glioblastoma microenvironment. Also, do the glioblastoma‐activated pericytes detach from the blood vessels and suffer a phenotypic switch? From a drug development perspective, pericytes provide a central cellular target with a stereotyped molecular repertoire and responses to signals. Nevertheless, as pericytes have important physiologic functions, what will turn out to be more challenging will be to limit deleterious pericyte functions while preserving the healthy ones. Additionally, it will be important to study whether pericytes'‐induced immunosuppression is important in other cancers.

## Other Perivascular Cells

The method of pericyte isolation used by Valdor et al. (2017) could result in the presence of other perivascular cells in these cultures. To avoid this, isolation by sorting based on several molecular membrane markers would be preferred. Although pericytes are defined by their anatomical perivascular position, not all perivascular cells are pericytes. Several cells that share molecular markers with pericytes have been described as perivascular: that is macrophages 74, 75, adventitial cells 76, smooth muscle cells 60, and fibroblasts 77. Even well‐established pericytic markers, PDGFR*β* and NG2, can be expressed in other cell types in certain pathophysiological settings. For instance, PDGFR*β* is a known marker of fibroblasts in the central nervous system 77, 78. NG2 proteoglycan can be expressed in macrophages 79, and pericytes not expressing NG2 were also described 38. Although none of brain pericyte markers are specific, when used in combination they distinguish pericytes from other cell types. Also, the combination of immunolabeling of the vascular basal lamina with pericyte molecular markers will confirm the exact nature of those cells. Recently, new molecular markers were described for pericytes, such as Tbx18 24, Gli1 80, and others. Future studies should clarify whether the perivascular population of cells activated by glioblastoma cells to produce immunosuppression in the brain in vivo are pericytes. Additionally, it will be interesting to explore the role of different perivascular cell populations in immunosuppression as well as tumor growth in the glioblastoma microenvironment.

Pericytes' capacity to form several cell types is well known; the general consensus holds that pericytes behave as stem cells under certain conditions 3, 24, 25, 26, 27, 80, 81. A recent study showed that pericytes expressing NG2 proteoglycan are the cell of origin for mesenchymal tumors, such as bone and soft tissue sarcomas 82. It will be interesting to explore whether the same may happen in the glioblastoma. Recently, it has been shown by lineage tracing technologies that, in the central nervous system, glioblastoma stem cells form pericytes that support blood vessel function and tumor progression 83, 84. Future works should explore whether some of the glioblastoma malignant cancer cells derive from pericytes. Another interesting question that derives from this study is whether or not pericyte‐induced immunosuppression in the glioblastoma microenvironment is reversible upon removal of the tumor cells. Pericyte‐intrinsic changes may be reversible or not but are continuous reinforcing signals from the glioblastoma cells needed for pericytes' production of anti‐inflammatory cytokines? Thus, analyses of pericytes, after long time of exposure to glioblastoma cancer cells, should be performed in future experimental settings.

## Signals Produced by Brain Pericytes

In addition to functioning as stem cells, pericytes can also regulate the behavior of other stem cells, being an important component of stem cell niches in several organs 60, 85. During embryonic development, perivascular niches for hematopoietic stem cells have been also described the spleen 86, placenta 87, and fetal liver 85. In the adult bone marrow, it was recently demonstrated that CXCL12 derived from pericytes is essential for hematopoietic stem cell maintenance in this organ 60. Also, in the brain, perivascular niches are important to regulate neural stem cells 88. These studies suggest that perivascular cells provide an adaptive niches for stem cells at physiologic conditions. One interesting question is whether pericytes also are important cellular components of the niche for glioblastoma stem cells. The study by Valdor et al. (2017) demonstrates that glioblastoma‐activated pericytes release IL‐10 and TGF*β* 48. Nevertheless, it still remains poorly explored whether other factors produced by brain pericytes are important for the support of glioblastoma growth. Pericytes release a plethora of molecules, including growth factors and cytokines 89, 90, 91, 92, 93, 94, 95, 96, 97, 98. Which molecules produced by brain pericytes are important during glioblastoma development remains to be elucidated. In addition to transcriptomic and single cell analysis, genetic mouse models will help to address this. For instance, using pericyte‐specific inducible CreER drivers crossed to cytokines‐floxed mice, specific cytokines could be deleted genetically at different time points specifically from pericytes in the brain, and glioblastoma progression could be analyzed. Thus, future studies should reveal whether there are common cellular and molecular mechanisms to form normal and glioblastoma stem cell niches in the brain.

## Conclusion

The study by Valdor and colleagues reveals a novel important role of pericytes in the glioblastoma microenvironment. Nevertheless, our understanding of pericytes biology in the brain still remains limited, and the complexity and interactions of different cellular components of the brain microenvironment during glioblastoma progression should be elucidated in future studies. An enormous challenge for the future will be to translate the research from experimental models into humans. Whether tumor cells during human cancer development promote the same immunosuppressive phenotype in human pericytes in the brain remains to be determined. Improving the availability of human glioblastoma samples will be essential to reach this objective.

## Conflict of Interest

The authors indicate no potential conflict of interests.
